# Comparative evaluation of direct and digital measurements of peri-implant defects using CBCT, ultrasound, and intraoral scanning

**DOI:** 10.1093/dmfr/twag006

**Published:** 2026-01-21

**Authors:** Mahmure Ayşe Tayman, Kıvanç Kamburoğlu, Esra Ece Çakmak

**Affiliations:** Department of Periodontology, Faculty of Dentistry, Ankara Yıldırım Beyazıt University, Ankara, 06010, Turkey; Dentomaxillofacial Radiology Department, Faculty of Dentistry, Ankara University, Ankara, 06500, Turkey; Arel Oral and Dental Health Clinic, Etimesgut, Ankara 06793, Turkey

**Keywords:** peri-implant defect, CBCT, ultrasound, intraoral scanner, diagnostic accuracy

## Abstract

**Objectives:**

To compare the measurement accuracy of cone-beam computed tomography (CBCT), high-resolution ultrasound (US), and intraoral scanning (IOS) with the gold-standard direct method in the measurement of peri-implant bone defects.

**Methods:**

Forty standard-threaded and 38 aggressive-threaded (Aggressor) implants—identical in diameter and length (4.3/10 mm) but differing in macro-thread design—were placed into bovine rib bones *in vitro*. Dehiscence, 2/3-wall, and 4-wall (circumferential) defects were prepared around the implants. Each defect was measured for maximum width, depth, and height using CBCT, US, IOS, and direct manual measurement. Analyses were performed using the general linear model (ANOVA). A *P*-value < .05 was considered statistically significant.

**Results:**

Intra-operator and inter-operator agreement showed high reliability (Gage R&R below 10%). For maximum width, defect type (*F* = 894.81, *P* < .001), method (*F* = 6.76, *P* < .001), and implant type (*F* = 5.39, *P* = .021) were significant. For maximum depth, defect type (*F* = 861.12, *P* < .001) and method (*F* = 3.39, *P* = .018) were significant. For maximum height, method (*F* = 12.62, *P* < .001) and defect type (*F* = 38.91, *P* < .001) were significant. The model demonstrated high explanatory power for width (*R*^2^ = 75.9%) and depth (*R*^2^ = 76.6%) measurements but lower for height (*R*^2^ = 20.7%). CBCT provided the most consistent results relative to direct measurements, followed by US, whereas IOS showed greater deviations.

**Conclusions:**

CBCT showed the highest agreement with direct measurements, followed by US, while IOS exhibited greater variability. Defect type and measurement modality were the primary determinants of accuracy. These findings indicate that CBCT and US can be considered reliable tools for assessing peri-implant bone defects.

## Introduction

Peri-implantitis is a chronic inflammatory disease characterized by progressive peri-implant bone loss, compromising implant longevity. Diagnosis is based on clinical parameters and radiological findings. Although periodontal probes are routinely used chairside as primary clinical tools, their reliability remains controversial. The circular and parallel orientation of peri-implant fibres, along with their weak hemidesmosomal attachment, may allow deeper probe penetration into peri-implant tissues.^1^ Furthermore, probe angulation, direction, applied pressure, and prosthetic emergence profile may influence diagnostic accuracy.

According to the World Workshop on the Classification of Periodontal and Peri-implant Diseases and Conditions, peri-implantitis is defined as bleeding and/or suppuration on gentle probing (0.15 Ncm), probing depth (PD) ≥ 6 mm, and progressive bone loss (preferably referenced from baseline radiographs) or ≥ 3 mm bone level apical to the most coronal part of the intraosseous implant component.^2^ However, peri-implant bone loss may occur without clinical signs of inflammation, and deep PD values can sometimes be observed in the presence of thick crestal mucosa without disease. In cases where PD < 6 mm, periapical radiography is recommended to verify mucositis. Clinical studies validated the predictive value of PD in diagnosing peri-implantitis, reporting that each additional 1 mm increase in PD doubles the probability of diagnosis.^3^

The macrogeometric properties (diameter, length, thread) of a dental implant, together with its surface properties and material, are critical for primary stability and osseointegration at the implant-bone interface. Surgical technique, loading time, and bone quality are also crucial for successful implantation. Implants with aggressive (sharp and deep) threads have limited indications for immediate implantation in soft and low-density bone (maxillary posterior regions, osteoporotic patients, areas of weak residual bone), and their primary stability is higher than that of non-aggressive, standard-threaded implants.^4^

Radiographic analysis is essential for accurate peri-implantitis diagnosis and treatment planning. Ideally, reference bone levels of ≥3 mm should be derived from baseline documentation. In routine dental practice, intraoral periapical radiography is the most commonly preferred modality; however, the two-dimensional nature of the technique and projection geometry issues limit its diagnostic capacity, resulting in underestimation of peri-implant bone loss. Studies have shown that periapical radiographs underestimate bone loss severity by about 1 mm,^5^ and the difference between periapical and intra-surgical bone assessments ranges between 1 and 2 mm.^6^ To accurately determine defect configuration—which is important for treatment planning—three-dimensional imaging modalities such as cone-beam computed tomography (CBCT) and conventional computed tomography (CT) have been recommended.^7^ Differences in sensitivity and specificity have been reported for intraoral X-ray, CT, and CBCT assessments depending on artefact presence and jaw location.^8^ CBCT, with its ability to visualize buccal and lingual bone using a limited field of view and acceptable radiation dose, has been suggested as a valuable diagnostic tool for the assessment of peri-implant bone defects. CBCT measurements have been shown to correlate acceptably with histologic measurements^9^ and with clinical defect morphology,^10^ providing submillimetre accuracy and high-quality images.^11^ Nevertheless, both under- and overestimation have been reported for CBCT measurements. Therefore, it is essential to justify its clinical use carefully due to radiation concerns. Moreover, CBCT accuracy may be reduced by bright metallic artefacts surrounding implants, producing radiolucent halos that obscure adjacent bone, necessitating special attention when assessing tissues around implants.^12^

Ultrasound (US) imaging has recently emerged as a promising noninvasive and radiation-free diagnostic tool for evaluating peri-implant bone defects.^13^ High-frequency US probes (>10 MHz) provide superior resolution for superficial structures.^14^ When imaging dense tissues such as bone, sound waves produce hyperechoic reflections, resulting in bright images. Newly developed compact intraoral probes have increased the versatility of US. Several studies have evaluated intraoral US probes for anatomical assessments around teeth^15^^,^^16^ and reported high reliability.^17^^,^^18^ Measurement errors within 0.6 mm have been considered clinically acceptable.^19^ Additionally, Doppler US has demonstrated a correlation between tissue perfusion patterns and peri-implant health/disease status.^13^^,^^20^ Hence, portable, user-friendly high-resolution US probes may serve as valid and effective tools for monitoring dental implants.

Intraoral scanners (IOS) represent another digital tool increasingly used in diagnostic and surgical workflows.^21–25^ Although IOS does not visualize subsurface bone structures, it provides high-resolution 3D surface models enabling quantitative assessment of exposed implant components and peri-implant bone morphology. Recent investigations have shown that IOS can detect changes in peri-implant soft and hard tissue contours, measure exposed threads, and support evaluation of defect dimensions once the lesion becomes clinically accessible.^23^^,^^24^ IOS may also be valuable intraoperatively for documenting defect morphology during peri-implant surgery and monitoring volumetric changes after regenerative procedures. These capabilities make IOS a potentially useful adjunctive method for assessing peri-implant defects,^21^^,^^24^ particularly when combined with other imaging modalities. IOS was included in the present study to evaluate its accuracy relative to CBCT, US, and direct measurements.

Accurate preoperative and intraoperative evaluation of peri-implant bone defects is critical for developing appropriate treatment strategies. Monje et al.^26^ classified peri-implant defects based on the presence and position of adjacent bony walls as intrabony, supracrestal, and combined intrabony–supracrestal types, with subcategories of dehiscence, 2/3-wall, and circumferential (4-wall) defects. Considering possible differences among diagnostic techniques, defect type, and implant design, the aim of the present study was to compare the measurement accuracy of direct manual, CBCT, US, and IOS methods in measuring dehiscence, 2/3-wall, and 4-wall peri-implant bone defects according to defect type and implant design.

## Methods

In this *in vitro* study, a total of 80 implants—40 standard (diameter/length = 4.3/10 mm) and 38 aggressive (Aggressor, diameter/length = 4.3/10 mm)—were placed into bovine rib bones, which are considered to resemble the human jawbone. The implants were of hybrid design, featuring a conical apex, cylindrical neck, and a modified RBM (resorbable blasting media) surface (Implance, Trabzon, Türkiye). The two implant types differed in their macro-thread design, although they had identical dimensions (4.3 mm diameter, 10 mm length) and the same RBM surface. The “standard” implant featured a semi-aggressive hybrid thread pattern with a cylindrical coronal portion and a conical apex, characterized by moderate thread depth and less pronounced cutting edges. In contrast, the “aggressive” implant (Aggressor) presented deeper, sharper, and more widely spaced macro-threads designed to increase primary stability, particularly in low-density bone. These terms (“standard” and “aggressive”) follow the manufacturer’s classification and do not represent universally standardized terminology in implantology. To prevent ambiguity, the present study uses these definitions solely to distinguish the two macro-thread designs.

*A priori* power analysis (1-way ANOVA) was performed assuming a clinically meaningful difference of 1 mm and a standard deviation of approximately 1.5 mm, with a significance level set at α = 0.05. Based on these assumptions, we determined that evaluating 78 defects (implants) per diagnostic method would be sufficient to detect a 1 mm difference. Under these conditions, the statistical power of the analysis was calculated as 94.9%, indicating that the sample size was adequate to detect clinically relevant differences with high confidence.

### Preparation of implant sockets

Two separate surgical kits supplied by the manufacturer were used for the preparation of implant sockets. Seven bovine ribs were completely stripped of soft tissue and cut into 15-20 cm segments for the experiments. Because bone density may vary across different regions of each rib, sockets for standard and aggressive implants were prepared alternately at equal intervals to minimize regional bias. Osteotomies were performed using a physiodispenser (Woodpecker-Implant Air, Guilin, China). For standard implant placement, drilling was performed sequentially with a marking drill followed by 2.2, 3.3, 3.7, and 4.3 mm burs. For aggressive implants, a marking drill followed by 2.8, 3.7, and 4.3 mm burs was used, in accordance with the manufacturer’s protocol.

### Creation of peri-implant defects

**Figure 1.**
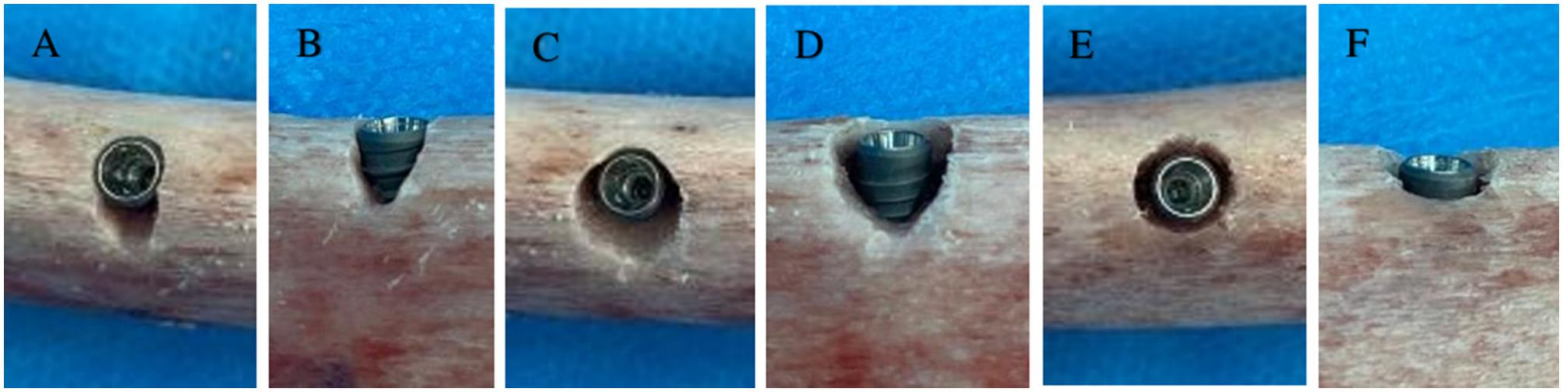
(A, B) Dehiscence defect (Type 1); (C, D) 2-3-wall defect (Type 2); (E, F) 4-wall (circumferential) defect (Type 3).

Before implant placement, three types of peri-implant bone defects were created around the implant neck using a high-speed, air-water-cooled diamond bur (Figure 1)^27^ as follows:

*Dehiscence-type defect (Type 1):* A narrow defect extending apically from the buccal marginal bone of the implant.
*Two- or three-wall intrabony defect (Type 2):* A combined defect including both buccal dehiscence and mesial/distal intrabony components.
*Four-wall circumferential defect (Type 3):* A complete intrabony defect surrounding the implant neck circumferentially.

### Direct (manual) measurements

Before defect measurements, a calibration session was conducted. Six defects were evaluated twice, with 1-h intervals between sessions. Repeated measurements were within ±1 mm in 85% of cases.

**Figure 2.**
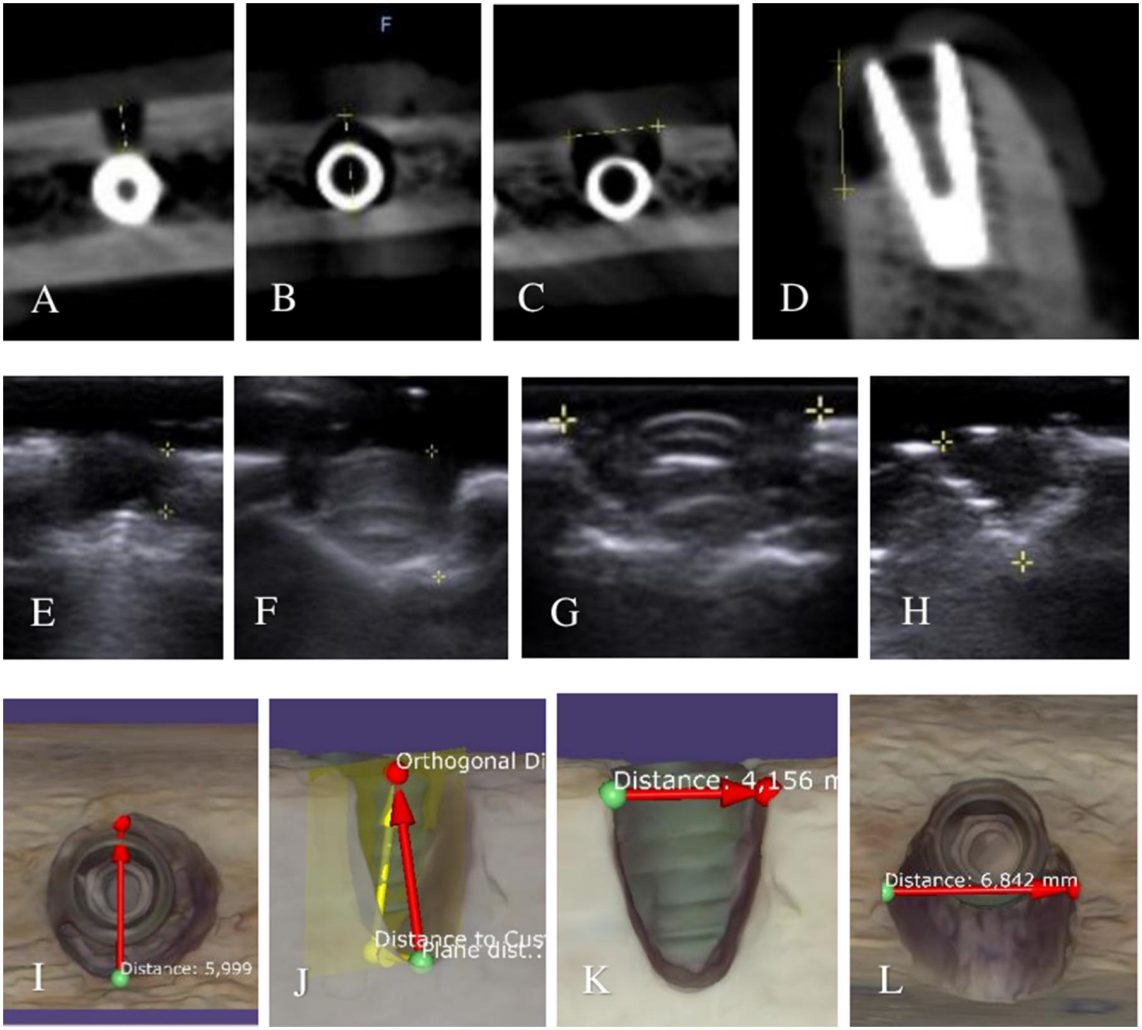
(A) Dehiscence—deepest; (B) four-wall—deepest; (C) 2-3-wall—widest; (D) dehiscence—highest; (E) dehiscence—deepest; (F) 2-3-wall—deepest; (G) 2-3-wall—widest; (H) four-wall—highest; (I) four-wall—deepest; (J) dehiscence deepest and highest; (K) dehiscence—widest; (L) 2-3-wall—widest.

Direct measurements were made using a calibrated periodontal probe (PCP-UNC-156, Hu-Friedy Mfg. Co., LLC, Chicago, IL, United States) and a digital caliper (Vernier, Altraco Inc., Sausalito, CA, United States) with a precision of 0.01 mm. Measurements of maximum width, depth, and height were recorded. Manual measurements were considered the “gold standard” for comparison. Metrics were defined as follows: *Maximum width:* largest mesiodistal distance. *Maximum depth:* deepest buccolingual distance. *Maximum height:* greatest coronoapical (vertical) distance (Figure 2).

All defects were measured twice by 2 experienced clinicians—an oral and maxillofacial radiologist and a periodontist—each with more than 10 years of experience in diagnosing and measuring peri-implant defects using manual, CBCT, and US methods. Measurements were repeated at 2-week intervals.

### CBCT imaging and measurement

**Figure 3.**
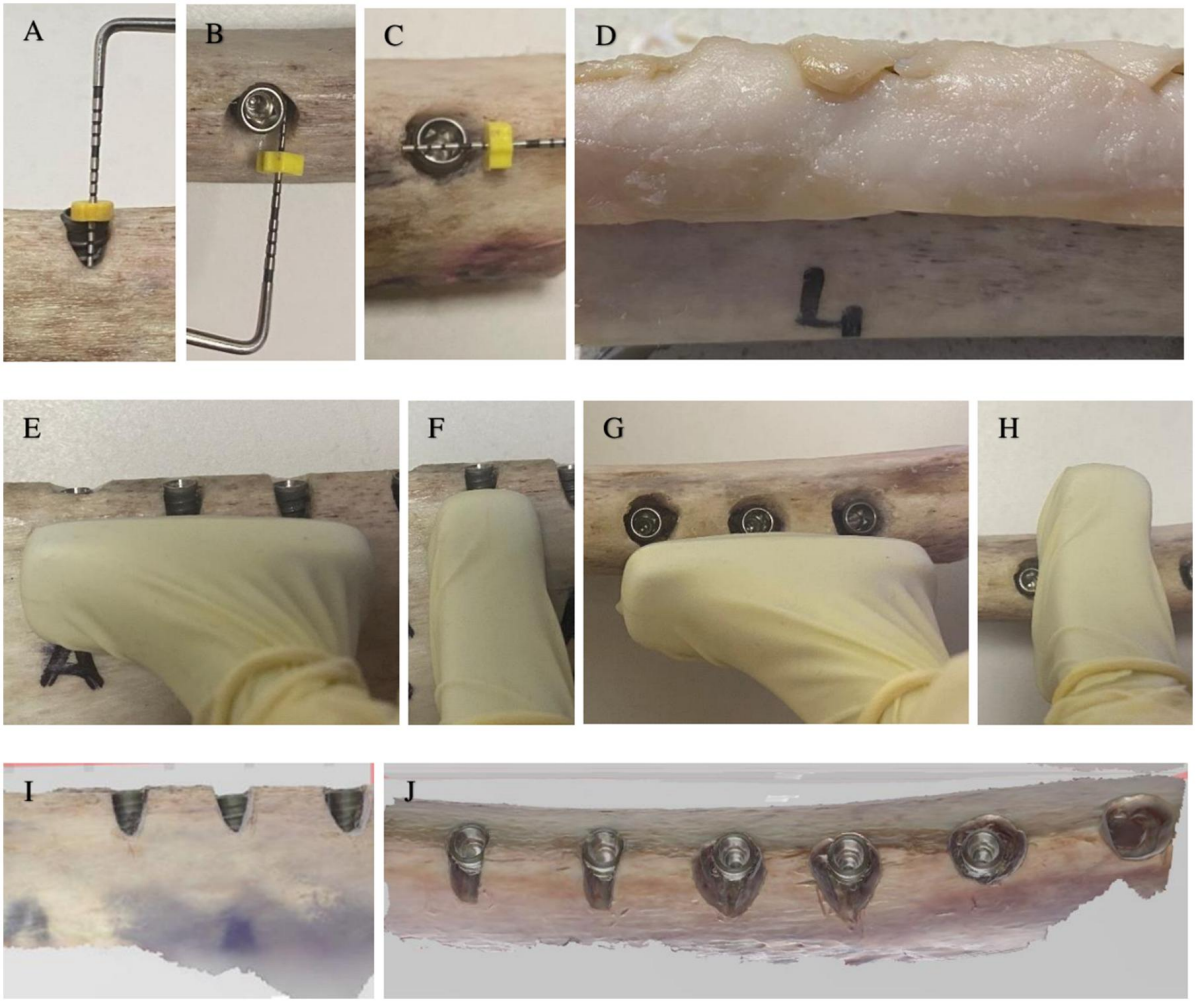
(A) Type 1—highest; (B) Type 2—deepest; (C) Type 3—widest; (D) bovine rib with soft-tissue-covered peri-implant defects; (E) Types 1 and 2—widest and deepest measured with buccal transverse probe; (F) Types 1 and 2—highest measured with longitudinal probe; (G) Type 3—widest and highest measured along occlusal plane; (H) Type 3—deepest measured buccolingually; (I-J) IOS scans imported into Exocad for measurement.

Before diagnostic imaging, soft tissues were positioned. Regular, unfragmented gingiva was removed from the cow’s jaw. Two large pieces of gingiva were used to cover each rib—one buccally and one lingually (Figure 3). Each rib was stabilized in a cardboard box. The imaging area was centred on the region of interest (ROI).

Dental volumetric images were acquired using a CBCT unit (Largev Smart 3D CBCT, Beijing, China) at 100 kVp, 6 mA, with a 12 × 8 cm FOV, 0.25 mm voxel size, and a multiplanar image acquisition time of 8-20 s using the default metal artefact reduction tool.

After a calibration session conducted on 3 defects (one from each defect type), CBCT measurements were performed by 2 observers (the same periodontist and radiologist who performed the manual measurements) using dedicated software. Observers prepared axial, panoramic, and cross-sectional images with a 1 mm slice interval and 1 mm thickness. Measurements were made as follows: *Height:* cross-sectional slices. *Width and depth:* axial slices.

All measurements were performed on a 2200 NEC MD213MG LCD monitor (NEC, Tokyo, Japan) with 2048 × 1536 resolution and 32-bit colour depth, twice, 2 weeks apart, in a darkened room, without time restrictions. The widest, highest, and deepest values were recorded.

### US imaging and measurement

Ultrasound (US) examinations were performed by the same researchers—who also conducted the manual and CBCT measurements—twice at 2-week intervals, using the ACUSON S2000 (Siemens, Munich, Germany) high-resolution ultrasound device. The ACUSON S2000 system consists of an ultrasound scanner and a specialized fixed transducer connected to a mechanical arm. The 14L5SP transducer (maximum frequency 14 MHz, average scan frequency 10 MHz, 15.4 cm width, 768 piezoelectric elements) acquires images at a maximum depth of 6 cm with an axial resolution of 0.09 mm and a lateral resolution of 0.16 mm. It provides imaging with a dynamic range of 30-90 dB and incorporates Dynamic TCE (Tissue Contrast Enhancement) technology. A gain-summing algorithm analyzes data to adjust brightness variations caused by transducer channel-to-channel effects.

Ultrasound examinations were performed in the longitudinal and transversal planes using a 14 MHz hockey-stick probe covered with ultrasound gel and a sterile sheath. The probe position was continuously adjusted to obtain adequate longitudinal and transversal images on the monitor. The transducer was positioned perpendicular to the implants to enable cross-sectional assessment of the lesion area.

Measurements were performed according to defect type: *Defect Type 1 (Dehiscence):* Height: longitudinal views. Width and depth: transverse probe positioning. *Defect Type 2 (2- or 3-wall):* Height: longitudinal views. Width and depth: transverse probe positioning. *Defect Type 3 (4-wall circumferential):* Width and height: transverse views. Depth: longitudinal probe positioning.

For *Defect Types 1 and 2*, the probe was positioned buccally and oriented transversely. The lesion was scanned continuously from the coronal to the apical aspect, and the widest and deepest areas were measured. The probe was then positioned longitudinally to measure the highest point of the defect along the implant threads.

For *Defect Type 3*, the probe was positioned at the crest and oriented from the mesial to the distal aspect, scanning from the buccal to the lingual aspect. The widest and highest points were recorded. Depth was measured by positioning the probe buccolingually and scanning from the mesial to distal aspect.

Defect tracking changes were visualized as bone (hyperechoic) → air (anechoic) → bone (hyperechoic). A hyperechoic line representing bone was observed beneath the anechoic area and was measured accordingly (Figure 3).

### Intraoral scanner imaging and measurement

Three-dimensional colour scans were obtained using an AI-assisted wired 3Shape TRIOS intraoral scanner (3Shape, Copenhagen, Denmark). A new “diagnostic” case was created for each scan, selecting the maxillary or mandibular jaw as appropriate. The scanning mirror was cleaned prior to use, and the “Start Scan” function was activated.

The bone surface and peri-implant defect areas were ensured to be clean, dry, and free of reflections. The specimen was stabilized to prevent movement during scanning. Scanning began from the occlusal surface of the implant site and continued systematically around the implant, including buccolingual and circumferential sweeps. Images were captured from multiple angles—top, lateral, and oblique—until complete coverage was obtained. If “scan errors” or “missing data” alerts appeared, the area was rescanned. Final scans were saved as STL (Standard Tessellation Language) files and stored in a dedicated folder for analysis.

These 3D models were imported into exocad DentalCAD 3.2 FR for measurement. Using the measurement tool, the following distances were recorded (by the same researchers who performed manual, CBCT, and US measurements, twice at 2-week intervals): mesiodistal maximum width, buccolingual maximum depth, and coronapical maximum height. Screenshots were taken after each measurement for documentation.

### Statistical analysis

Data analysis was performed using the general linear model (ANOVA). The effects of measurement method, implant type, and defect type were analyzed for each dimensional variable (width, depth, height). Statistical significance was set at *P* < .05.

## Results

### Reliability analysis

All measurements across all methods had total variation (Gage R&R) values below 10%, demonstrating high system reliability—indicating strong repeatability and consistency across operators. The measurement system was capable of distinguishing true differences between samples, and results were statistically consistent.

### Width measurements

**Figures 4.**
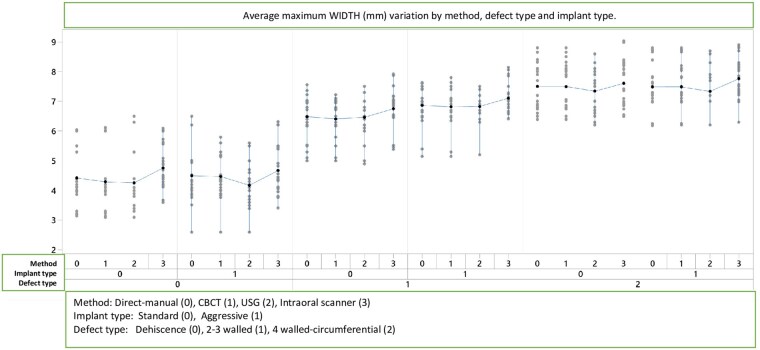
Average maximum width (mm) variation by method, defect type and implant type.

The linear model for maximum width measurements according to method, implant type, and defect type is presented in Table 1 and Figure 4. Significant differences were found among methods (*P* = .000), implant types (*P* = .021), and defect types (*P* = .000). The only significant interaction was between implant type × defect type (*P* = .025).

**Figures 5.**
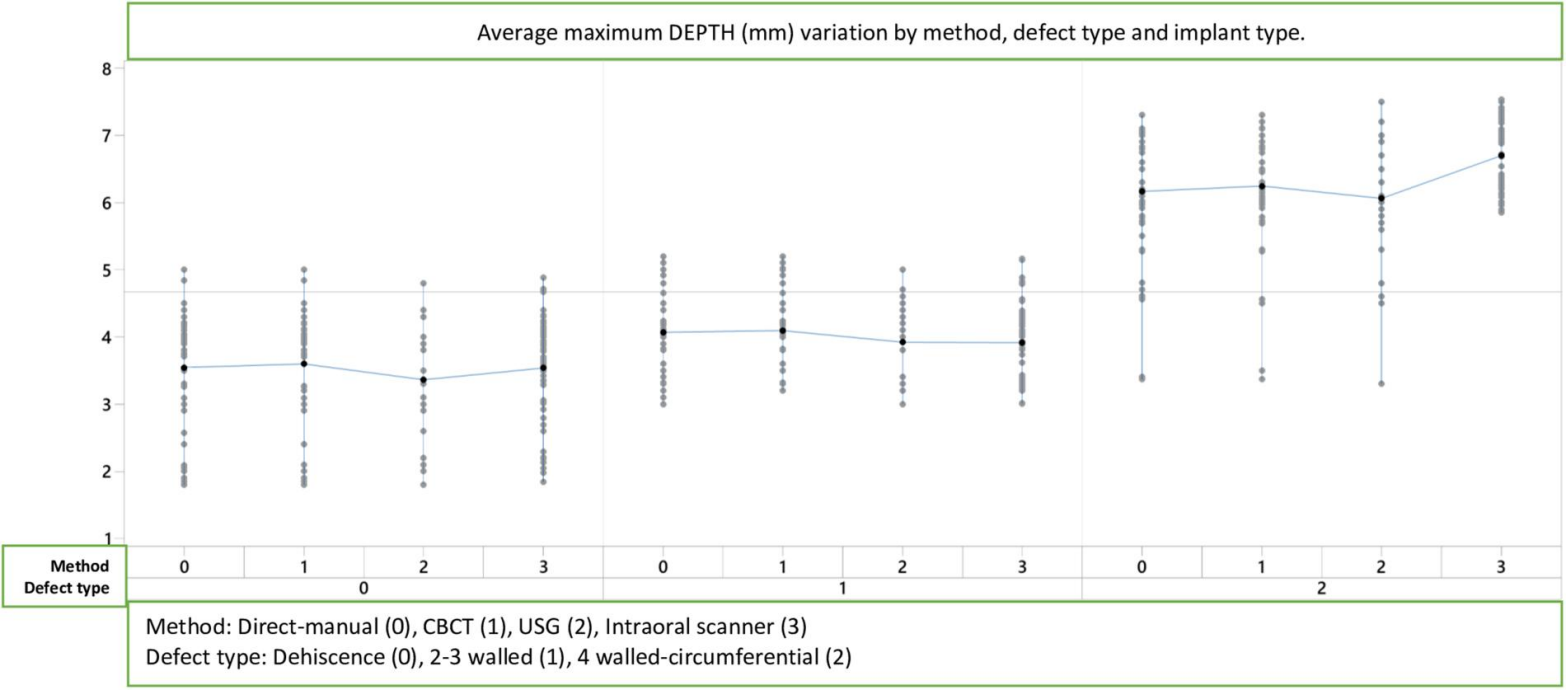
Average maximum depth (mm) variation by method, defect type and implant type.

**Figures 6.**
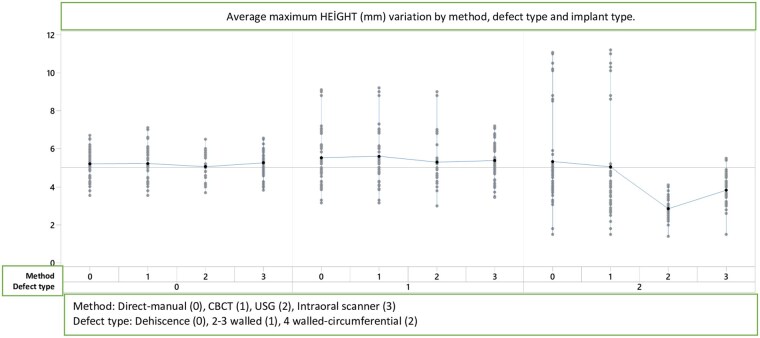
Average maximum height (mm) variation by method, defect type and implant type.

**Table 1.** General linear model: WIDEST, DEEPER, HIGHEST distance versus method, implant, and defect type.

Source	DF	Adj. SS	Adj. MS	*F*-value	*P*-value	Adj. SS	Adj MS	*F*-value	*P*-value	Adj. SS	Adj. MS	*F*-value	*P*-value
		**Maximum width**				**Maximum depth**				**Maximum height**			
Method	3	11.13	3.709	6.76	.000	5.09	1.696	3.39	.018	78.01	26.0022	12.62	.000
İmplant type	1	2.96	2.960	5.39	.021	0.80	0.802	1.60	.206	0.76	0.7644	0.37	.543
Defect type	2	982.18	491.088	894.81	.000	862.53	431.263	861.12	.000	160.37	80.1863	38.91	.000
Method * implant type	3	0.14	0.046	0.08	.969	1.82	0.607	1.21	.304	1.03	0.3426	0.17	.919
Method * defect type	6	1.08	0.179	0.33	.923	10.99	1.832	3.66	.001	100.21	16.7019	8.10	.000
İmplant type * defect type	2	4.10	2.048	3.73	.025	19.20	9.599	19.17	.000	16.71	8.3555	4.05	.018
Error	608	333.68	0.549			304.50	0.501			1251.04	2.0610		
Lack-of-fit	6	0.81	0.134	0.24	.962	2.33	0.389	0.77	.590	3.39	0.5649	0.27	.950
Pure error	602	332.87	0.553			302.16	0.502			1247.66	2.0760		
Total	625	1385.10				1299.91				1577.47			
Model summary		*S*	*R*-sq	*R*-sq(adj)	*R*-sq(pred)	*S*	*R*-sq	*R*-sq(adj)	*R*-sq(pred)	*S*	*R*-sq	*R*-sq(adj)	*R*-sq(pred)
		0.740822	75.91%	75.24%	74.44%	0.707683	76.58%	75.92%	75.12%	1.43563	20.69%	18.47%	16.26%

CBCT produced values closest to the direct (manual) measurements, whereas IOS demonstrated the greatest deviation. CBCT and US were the most reliable methods for width measurements. Results showed that method, implant type, and defect type significantly affected width values.

### Depth measurements

The linear model for maximum depth measurements by method, implant type, and defect type is presented in Table 1 and Figure 5. Significant differences were found for measurement method (*P* = .018) and defect type (*P* = .000). Implant type did not have a significant effect on depth measurements (*P* = .206). Significant interactions were identified for method × defect type (*P* = .001) and implant type × defect type (*P* = .000).

CBCT and US provided values that were most comparable to direct measurements, indicating superior reliability for depth evaluation. IOS measurements displayed greater variability. Among all methods, CBCT exhibited the highest accuracy, particularly for deeper defect configurations. Differences were most prominent across defect types, especially in four-wall (circumferential) defects.

### Height measurements

The linear model for maximum height measurements according to method, implant type, and defect type is presented in Table 1 and Figure 6. Significant differences were found for measurement method (*P* = .000) and defect type (*P* = .000). No significant effect was observed for implant type (*P* = .543). Significant interactions occurred between method × defect type (*P* = .000) and implant type × defect type (*P* = .018).

CBCT and US again produced values closest to direct measurements, confirming their reliability for assessing defect height. IOS measurements showed greater discrepancies than the other modalities. CBCT demonstrated the closest approximation to gold-standard measurements, while the most pronounced differences were observed for height values in the defect models.

## Discussion

In the present research, we evaluated three common types of peri-implant bone defects using four different measurement methods in three dimensions (width, depth, and height). CBCT demonstrated the closest agreement with direct measurements, followed by US with clinically acceptable accuracy, whereas IOS showed the greatest deviation. Among all factors, defect type was the strongest determinant influencing measurement results across all methods (*P* < .001), whereas implant type produced a significant difference only in width measurements.

The diagnostic accuracy of CBCT was found to be influenced by study design and defect characteristics.^7^ Additionally, implant material composition may affect its precision.^28^ The accuracy of CBCT is impaired by artefacts caused by metallic implants, where bright artefacts around the implants lead to a radiolucent shadow surrounding them.^12^ CBCT demonstrated high sensitivity in diagnosing peri-implant defects in nearly all included studies, with an average discrepancy of less than 1 mm from the true value of the defect.^7^^,^^10^^,^^28–30^ Insua et al.^10^ found that peri-implant bone defect morphology assessed by CBCT matched clinical defect configurations with 100% accuracy. Similarly, in the present study, CBCT results closely correlated with gold-standard direct measurements, with underestimation values below 0.5 mm. In another study, although CBCT demonstrated high sensitivity in detecting peri-implant bone defects, linear measurements of defect height and width were underestimated by an average of <1 mm.^28^

*Ex vivo* studies have demonstrated good sensitivity and specificity for both circumferential and intrabony defects, but lower values for dehiscence defects.^10^^,^^30^^,^^31^ However, when compared to clinical evaluation, CBCT tends to yield lower values for defect depth and height.^27^ Similarly, mean differences of approximately 1 mm have been reported between CBCT and histological measurements of defect height.^10^ In our study, the model’s explanatory power was high for width (*R*^2^ = 75.9%) and depth (*R*^2^ = 76.6%) but lower for height (*R*^2^ = 20.7%). The discrepancy was most evident in tall, narrow, four-wall circumferential defects, suggesting that defect type and measurement method are key determinants of morphometric accuracy. An *in vitro* study also reported that CBCT provided the highest image quality and accurately delineated peri-implant defects in all three planes without distortion.^11^

*In vivo* animal studies have shown good correlation between CBCT and histologic analysis, with an average discrepancy of approximately 0.5 mm for intrabony and supracrestal defects in experimental peri-implantitis models.^9^ Therefore, incorporating CBCT into standard diagnostic protocols for peri-implantitis should be justified only when clearly clinically indicated, taking radiation dose and economic considerations into account.^32^ Routine CBCT use for diagnosis and severity assessment is not recommended. Nevertheless, its application may be warranted for detailed evaluation of defect configuration, implant position, preoperative diagnosis, and surgical planning when two-dimensional systems fail to provide sufficient information. CBCT is also valuable when neurovascular complications are suspected after implant placement. Although radiographic assessment remains essential for visualizing the extent and configuration of bone loss, it may underestimate true defect size in peri-implantitis.

When metal is present in the area to be scanned, images are prone to artefacts. An artefact is any distortion or error in the image that is unrelated to the subject, and it is a major cause of decreased image quality; in some cases, the artefact may render the image useless.^12^ Some artefacts are produced due to a phenomenon referred to as beam hardening. When the X-ray beam travels through an object, low-energy photons are absorbed more than high-energy photons; this phenomenon, known as beam hardening, is produced by high-density objects (e.g., titanium implants).^12^ Exposure conditions can contribute to artefact formation by influencing photon energy; several studies have recommended imaging techniques with high kVp to reduce beam hardening.^12^ Optimal selection of CBCT acquisition protocols for peri-implant bone assessment is crucial, balancing diagnostic image quality and acceptable radiation dose. In endodontics and periodontics, evidence suggests that acceptable image quality can be achieved with optimal kVp, mA, and exposure time settings. Numerous factors related to technical acquisition parameters—including field of view (FOV), voxel size, and number of frames—directly influence the detection of small peri-implant defects. It is possible that the selection of 100 kVp and the use of a default metal artefact reduction tool in the present study facilitated better observer performance by decreasing beam-hardening artefacts. Further studies are needed to fully understand the effect of CBCT settings on peri-implant assessment.

Intraoral ultrasound (US) has been used for various periodontal diagnostic purposes.^33^^,^^34^ Periodontal structures imaged with a 24-MHz intraoral probe showed good correlation with direct clinical measurements.^16^ Due to its noninvasive and radiation-free nature, US has been proposed as a reliable tool for gingival thickness measurements compared with direct probing.^35^ Owing to the significant acoustic contrast between soft tissue and bone, US can effectively assess alveolar bone level and gingival thickness. Previous reports have shown high interexaminer reliability for these parameters,^17^^,^^18^ consistent with our results. In the present study, high-resolution US provided diagnostic accuracy comparable to CBCT and exhibited strong agreement with direct measurements. This may be attributable to the fact that our observers were experienced in periodontal tissue and bone measurement using US, which requires expertise.

Supporting these findings, previous studies demonstrated good to excellent correlations between US and direct measurements of soft tissue height, gingival thickness, and alveolar bone level.^16^ The difference between repeated alveolar bone-level measurements using US was reported to be less than 0.65 mm, showing comparable accuracy to clinical attachment level (CAL) assessments in periodontitis diagnosis.^36^ According to our data, underestimation was more pronounced in four-wall defects, where height values were lower in narrow and deep peri-implant defects. Nevertheless, US remains a promising diagnostic adjunct for preoperative assessment of peri-implant bone defect type and severity before surgical intervention. Integrating US into routine periodontal and peri-implant evaluations could enhance diagnostic precision, optimize treatment planning, and potentially reduce implant loss risk, thereby improving patient care. Moreover, US may supplement radiographs in assessing facial bone thickness and identifying dehiscence defects.^37^ Cadaver studies have shown that CBCT has limited resolution in differentiating thin facial bone (<1 mm) in the anterior maxilla.^38^ Thus, US may serve as a practical chairside diagnostic tool for assessing alveolar bone levels prior to periodontal or peri-implant surgery. The diagnostic accuracy and versatility of US depend on observer performance, the US probe used, and patient-related factors. Additionally, acoustic impedance may vary depending on the patient and the ultrasonic characteristics of tissues. Patient-related factors were not a concern in the present *in vitro* study.

In our study, IOS exhibited the largest deviation from direct measurements in all conditions. While intraoral scanners offer significant advantages and broad clinical applications in digital dentistry, the incremental image-acquisition process and “stitching” of multiple images can accumulate alignment errors, leading to measurable deviations.^39^ Scanning technique, operator proficiency,^40^ scanning strategy,^23^^,^^24^ and calibration errors are also known to significantly influence accuracy. Researchers have shown that digital model precision depends on the scan’s starting point,^41^ and avoiding vertical rotation during scanning improves accuracy.^25^^,^^42^ Studies evaluating multiple IOS systems have confirmed that scanning strategies and data-capture mechanisms affect accuracy.^43^^,^^44^ In this study, scanning began sequentially from the occlusal platform of the implant. It has been reported that initiating scans from complex occlusal surfaces and maintaining a sequential pattern minimizes stitching errors.^25^ However, reflective metal implant surfaces may have negatively affected scanning accuracy.^45^ The literature also indicates that scanning may be less reliable in patients with misaligned or crowded teeth, deep cavities, or reflective orthodontic brackets.^41^^,^^46^ Nevertheless, the resulting deviations were considered clinically acceptable for orthodontic diagnostics and treatment planning.^47^^,^^48^ However, occlusal contact recordings—important in procedures such as interproximal enamel reduction—may require higher accuracy.^49^ Although IOS showed the greatest deviation from direct measurements in our results, particularly in height measurements of narrow, deep four-wall defects, discrepancies for dehiscence and 2/3-wall defects remained within clinically acceptable limits. The deepest area of the defect is in close proximity to the implant; the presence of significant artefacts and radiolucency adjacent to the implant appears to make accurate defect detection difficult.^30^^,^^50^ A similar situation may apply to IOS due to metallic surface reflection. Given that IOS captures surface topography rather than subsurface structures, it may be more suitable for intraoperative rather than routine diagnostic use. In particular, IOS may be a valuable tool for intraoperative assessment during regenerative or grafting procedures, although further research is needed to validate its application in this context.

The implants used in the study had the same length, diameter, and surface (RBM) characteristics. Implants with a diameter of 4 mm and a length of 10 mm are among the most commonly preferred sizes for both surgical feasibility and clinical success. The standard implant has a hybrid design with a conical apex and a cylindrical coronal surface. The neck is straight and cylindrical, and the threads become more aggressive toward the apex, resulting in a semi-aggressive design. The aggressor implant, on the other hand, has a hybrid design with enhanced aggressive, macro, deep, and sharp thread patterns. Our results indicated a significant difference between implant types in width measurements but not in depth and height measurements. Differences in thread design may have affected results in defects with a greater number of bony walls, altering metallic scattering and reflection rates. In addition to thread design, surface roughness, different diameters and lengths, and different materials can also cause variations in defect visualization.

As emphasized, defect configuration plays an important role in the decision-making process in peri-implantitis management.^27^ This study evaluated the most common intrabony two-third-wall defects (55%) and intrabony dehiscence defects (16%), and the least common circumferential four-wall defects (3%). Defects with supracrestal components or supracrestal–intrabony combinations could also be evaluated using different imaging modalities in future studies.

Although US imaging is a real-time, non-ionizing, and well-tolerated method, its main drawback lies in variability in image acquisition due to operator- and patient-dependent factors. Patient-related factors such as body weight, adiposity, gender, and age-related reductions in tissue water concentration may negatively affect image quality; however, these were not relevant to our *in vitro* setting. Additionally, researchers who acted as observers in the present study were experienced in peri-implant defect measurement using all techniques applied.

Traditional radiographic techniques, including periapical and panoramic radiographs, provide two-dimensional representations. CBCT, by contrast, offers three-dimensional visualization and provides more realistic measurements than conventional radiographs. However, CBCT exposes patients to ionizing radiation and is not available in every clinical setting. Moreover, beam-hardening artefacts caused by dental implants and restorative materials may impair image quality and measurement accuracy.

Despite its promising capabilities, the current implementation of IOS for imaging periodontal defects still faces several challenges. Due to its reflective properties, imaging deeper structures may be problematic. For periodontal defects to be scanned with IOS, the area must be free of overlying soft tissue, requiring scanning with intraoperative flap reflection as a supplementary technique. This may assist clinicians in diagnosis, grafting decisions, and follow-up. Additionally, similar to US, IOS scanning is operator dependent.

Finally, the *in vitro* design of this study constitutes a limitation, as it cannot fully replicate the complexity of real clinical conditions. Bovine rib models commonly used in *in vitro* studies resemble the human jaw in contour and size and possess similar cortical–cancellous bone.^50^ However, surgically created defects often have sharper boundaries than those observed clinically. In clinical settings, defect boundaries are more diffuse and less distinct.^7^  *In vivo* image quality is affected by numerous additional factors, including limited intraoral space, moisture, patient movement, gag reflex, mucosal translucency, anatomical variations, and the number and density of metallic elements within the field of view.

## Conclusions

In this *in vitro* study comparing the measurement accuracy of CBCT, US, and IOS against gold-standard direct measurements, CBCT demonstrated the highest overall agreement, followed closely by US, which showed clinically acceptable accuracy across most defect types. IOS exhibited greater variability, particularly in narrow and deep four-wall defects, although deviations for dehiscence and 2/3-wall defects remained within clinically acceptable limits. Defect configuration and the measurement modality—rather than implant design—were the primary determinants influencing dimensional accuracy. These findings suggest that CBCT and US can serve as reliable tools for the assessment of peri-implant bone defects when clinically indicated, whereas IOS may be better suited as an adjunctive or intraoperative tool rather than a primary diagnostic modality.

## Advances in knowledge

The present study advances current knowledge by validating the diagnostic accuracy of CBCT and high-resolution ultrasonography as reliable, non-invasive alternatives to direct measurement for assessing peri-implant bone defects. It also highlights the potential role of intraoral scanning as a supplementary digital tool for clinical evaluation.
